# Analysis of Hepatitis E Virus-Like Sequence in Chimpanzee

**DOI:** 10.5812/hepatmon.19473

**Published:** 2014-09-01

**Authors:** Chenglin Zhou, Wang Li, Shixing Yang

**Affiliations:** 1 Department of Microbiology, School of Medical Science and Laboratory Medicine, Jiangsu University, Zhenjiang, China

**Keywords:** Hepatitis E Virus, Chimpanzee, Multilocus Sequence Analysis

*Dear Editor,*

**Figure 1.**
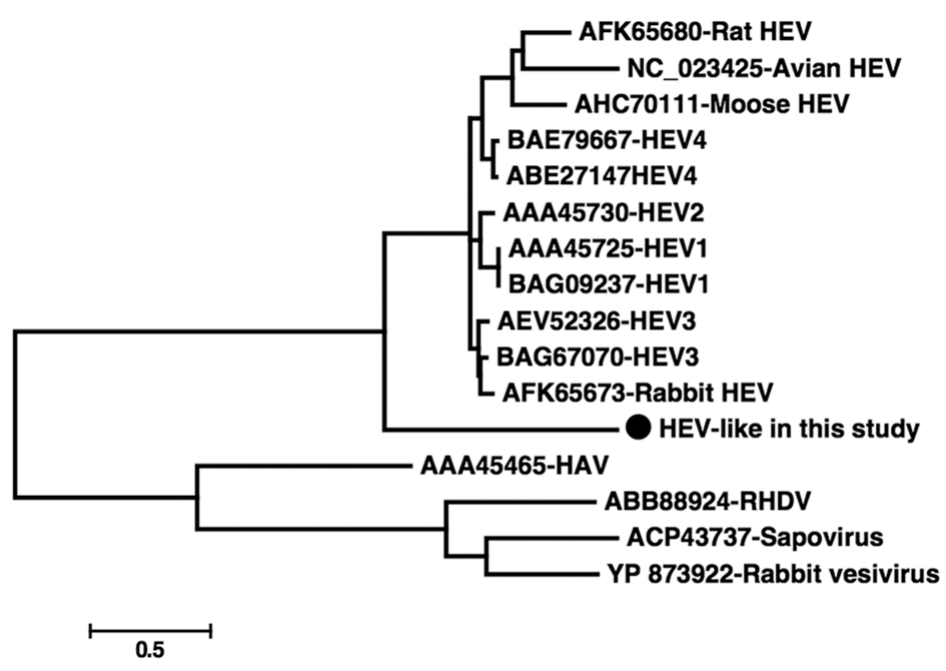
Phylogenetic Analysis of the HEV-Like Sequence in the Present Study and the Reference Sequences from GenBank The phylogenetic tree was produced with the amino acid sequence alignments of the sequence in the present study and another 15 reference sequences, using the maximum-likelihood method with Mega 5 software. The sequence identified in the current study is marked with a black circle.

Hepatitis E virus (HEV), a member of the genus Hepevirus, is a non-enveloped virus with a positive stranded RNA genome that is approximately 7.2 kb in length (1). It has been hypothesized that zoonosis is involved in the transmission of HEV (2, 3). Hepatitis E virus antibodies or genes have been reported to exist in many species of mammals, including monkeys (4, 5). Recently, divergent HEV strains has been discovered in different animals, including rats (6), mouses (7), and rabbits (8), which suggests that more animal species could be the reservoir of HEV. In the present study, we analyzed a HEV-like sequence, which was found by chance during the discovery of RNA virus in fecal samples of Chimpanzee from a zoo in China. Briefly, we extracted total RNA from the fecal suspension and performed reverse transcription using a primer containing a fixed sequence followed by a randomized octomer at the 3′ end. A single round of DNA synthesis was then performed using Klenow fragment polymerase. Twenty cycles of PCR amplification of nucleic acids was then performed using primers consisting of fixed portions of the random primers. Then the PCR products were purified, cloned into T-vector and sequenced. The resulting sequences were searched in GenBank using BLASTx. Searching results showed that one 685 bp sequence had the highest sequence homology with HEV, sharing 45-58% sequence identities. Sequence analysis revealed that the putative amino acid sequence of this fragment included the whole RdRp domain, which contained 157 amino acids. Due to the high divergence of the sequence, multiple attempts to acquire longer sequences of this virus failed. In order to investigate whether this sequence is prevalent in the Chimpanzee population, a set of primers were designed according to the 685 bp sequence in the present study to perform PCR screening in 24 fecal samples collected from Chimpanzees at the same zoo and 13 fecal samples from another zoo in China. The primers were Chev1 [5’-TGTCCTCATGTCTGTCAGG-3’] and Chev2 [5’-AATCACATCTACCAACAGC-3’] for the first round of PCR, and Chev3 [5’-TGCCACGGTCCACCGATCG-3’] and Chev4 [5’-ATAGAACCACCGGCGTTG-3’] for the second round. This set of nested primers was designed to amplify a 154-nt segment. Our PCR screening results indicated that seven (29.2%) of the 24 fecal samples from the same zoo were positive for this HEV-like sequence, while none of the 13 samples from the other zoo were positive, which suggests that this virus strain was highly prevalent in the Chimpanzee population at the studied zoo. The seven positive samples were cloned and sequenced; results indicated that they shared > 99% identity over nucleotide level, suggesting they belonged to the same virus strains. In order to further identify the genetic relationship between the sequences of this study and other known HEV strains, we performed a phylogenetic analysis based on the predicted amino acid sequences in the current study and those related sequences retrieved from GenBank. The HEV sequences included those from well-known HEV genotypes 1-4 (from human or pig), rat HEV, mouse HEV, rabbit HEV, and avian HEV. The other four related virus sequences were also added as outgroups, including hepatitis A virus, sapovirus, rabbit vesivirus and rabbit hemorrhagic disease virus. Briefly, amino acid sequences were aligned using Clusta lW v2.0. Phylogenetic analysis was constructed using the Mega 5 software (http://www.megasoftware.net/). GenBank accession numbers of the sequences used as references in this analysis are shown in Figure 1. The sequences determined in the current study were deposited in GenBank; strain name being Nhev-Cb1 and the accession number KM407530. Our phylogenetic analysis indicated that Nhev-Cb1 clustered with the other HEV sequences, lying in a deep branch with high bootstrap value of 100 (Figure 1). Over the amino acid sequence level, Nhev-Cb1 shared 35-39% sequence identity with the other HEV species, and 29-32% sequence identities with the four reference sequences. Our phylogenetic results suggested that the virus in the present study was a novel type of HEV. Although the antibodies to HEV and human origin HEV genes were discovered in non-human primates (4, 5, 9), these animals are not considered as the natural reservoirs for HEV. In the present study, HEV-like sequences were detected in seven (29.2%) of 24 Chimpanzees at the studied zoo, suggesting that if the sequences are from real viral particles, the virus may be a new type of HEV using non-human primate as its natural host.
